# The provision of malaria services in border districts of four countries in Southern Africa: results from a cross-sectional community assessment of malaria border health posts

**DOI:** 10.1186/s12936-023-04687-z

**Published:** 2023-10-20

**Authors:** Mukosha Chisenga, Bongani Dlamini, Nyasha Mwendera, Paulo Maquina, Busiku Hamainza, José Franco Martins, Francisco Saute, Henrico Bock, Roly Gosling, Adam Bennett, Jennifer Smith, Immo Kleinschmidt

**Affiliations:** 1 Southern African Development Community Malaria Elimination Eight Secretariat, Windhoek, Namibia; 2 Ministry of Health, National Malaria Elimination Centre, Chainama Hospital College, Lusaka, Zambia; 3 National Malaria Control Programme, Ministry of Health, Luanda, Angola; 4 Manhiça Health Research Centre, Manhica, Mozambique; 5 Multidisciplinary Research Centre, University of Namibia, Windhoek, Namibia; 6 Malaria Elimination Initiative, Global Health Group, University of California, San Francisco, San Francisco, USA; 7 Faculty of Health Sciences, School of Pathology, Wits Research Institute for Malaria, University of the Witwatersrand, Johannesburg, South Africa; 8 MRC International Statistics and Epidemiology Group, Department of Infectious Disease Epidemiology, London School of Hygiene and Tropical Medicine, London, UK; 9 Department of Disease Control, London School of Hygiene and Tropical Medicine, London, UK; 10 PATH, Seattle, USA

**Keywords:** Malaria, KAP, Border health post, Elimination

## Abstract

**Background:**

The importation of parasites across borders remains a threat to malaria elimination. The Southern African Development Community Malaria Elimination Eight (E8) established 39 border health facilities on 5 key international borders between high and low-burden countries. These clinics aimed to improve access to prevention, diagnosis, and treatment of malaria for residents in border areas and for mobile and migrant populations who frequently cross borders. Studies were conducted in each of the four high-burden E8 countries (Angola, Mozambique, Zambia, and Zimbabwe) to evaluate malaria services in border areas.

**Methods:**

Cross-sectional surveys were conducted within 30 km of recently established E8 Border Health Posts. Structured questionnaires were administered to randomly selected respondents to assess malaria-related knowledge and behavior, access to malaria prevention, diagnosis and treatment of malaria, and risk factors for malaria associated with local and cross-border travel.

**Results:**

Results showed that most providers followed appropriate guidelines performing blood tests when individuals presented with fever, and that nearly all those who reported a positive blood test received medication. Lack of access to health care due to distance, cost or mistrust of the provider was rare. A minority of respondents reported not receiving timely diagnosis either because they did not seek help, or because they were not offered a blood test when presenting with fever. There was a high level of correct knowledge of causes, symptoms, and prevention of malaria. A majority, of border residents had access to primary prevention against malaria through either long-lasting insecticidal nets (LLINs) or indoor residual spraying (IRS). Cross border travel was common with travellers reporting sleeping outside without protection against malaria.

**Conclusions:**

The study demonstrated the importance of border health facilities in providing access to malaria services. Prevention needs to be improved for people who travel and sleep outdoors. Community health workers can play a key role in providing access to information, testing and treating malaria.

## Background

Malaria remains a major global health challenge with 241 million annual cases and 627,000 deaths recorded worldwide in 2020. The bulk of this disease burden falls on Africa with 95% of all malaria cases and all malaria deaths occurring in the World Health Organization (WHO) African Region [1]. Whilst there have been recent successes in eliminating the disease from their territory by some countries, no country in sub-Saharan Africa has achieved elimination to date.

In southern Africa some countries have reduced malaria transmission to levels low enough that elimination has become a realistic prospect [2]. Realizing the need for inter-country coordination in the pursuit of elimination, eight countries in southern Africa established the Elimination 8 (E8) initiative under the Southern African Development Community (SADC) in 2009 [2]. The E8 facilitates the harmonisation of malaria control and elimination strategies between member countries with the ambitious goal of eliminating malaria by 2030. The E8 comprises of two groups of countries: the southernmost so-called frontline four (Botswana, Namibia, South Africa, and Eswatini), where malaria incidence has been reduced to less than 5 cases per 1000 per year, and their northern neighbours, the so-called second-line four (Angola, Mozambique, Zambia, and Zimbabwe) where a high burden of transmission persists with over 25 million cases reported in 2020 [3], see Table 1. In the frontline countries large declines in annual malaria cases were achieved from 2000 to 2015, but progress has plateaued in recent years with a resurgence of cases seen in outbreak years such as 2017 [2]. In second line countries the total number of malaria cases has increased since 2015 [1, 4].

**Table 1:** Malaria cases and annual incidence per 1000 population in E8 countries in 2018

	Frontline				Second line			
	Botswana^a^	Eswatini^a^	Namibia^a^	South Africa^a^	Angola^a^	Mozambique^a^	Zambia^a^	Zimbabwe^b^
Population at risk	1,494,401	318,156	1,943,338	5,779,252	30,809,787	29,496,009	17,351,714	14,042,504
Malaria cases	585	656	36,740	10,789	5,150,575	9,292,928	5,039,679	264,283
Incidence per 1000 p.a.	0.39	2.06	18.91	1.87	167.17	315.06	290.44	18.82

^a^WHO: World Malaria Report 2019, Annex 3 H. Geneva: World Health Organisation [1]

^b^National Malaria Control Programme, Zimbabwe

A major challenge facing countries striving for malaria elimination is the cross-border movement of people between countries with a high malaria burden to countries with low malaria transmission [5, 6]. The southern African region is characterised by a high degree of inter-connectedness between countries through economic and cultural ties, population movement as well as shared vector ecologies [7, 8]. The higher level of malaria transmission in second-line countries serves as a perpetual reservoir of infection that is imported into the four frontline countries [9]. To address this impediment towards achieving elimination, the Elimination 8 Strategic Plan of 2015 to 2020 had as one of its primary objectives the need to reduce cross-border malaria transmission [10]. The rationale was that importation of malaria could be mitigated by expanding access to malaria services for residents of remote, often underserved border districts as well as cross-border mobile and migrant populations (MMPs). Thus the E8 in collaboration with National Malaria Control Programmes (NMCPs) and other partners, established 46 border health facilities on five key international borders between high and low transmission districts of E8 countries in 2017 [2].

To better understand the malaria situation in border areas and the role that border posts and other health facilities in border areas have in providing malaria services, a descriptive study was undertaken to determine amongst border populations their access to malaria prevention, diagnosis and treatment; knowledge, perceptions and behaviour related to prevention and treatment-seeking; and patterns of travel.

This paper reports on the findings of the study conducted in the four second-line countries (Angola, Zambia, Zimbabwe and Mozambique).

## Methods

The study reported in this paper was part of a larger investigation, the E8 border post impact evaluation study, which was undertaken in seven countries in the E8 region. The methods and objectives differed between the three front-line countries (Namibia, Botswana, South Africa) in which it was conducted, and the four second-line countries. In this paper, only the results of the study conducted in second-line line countries are reported. Related studies in frontline countries were completed later and will be reported separately. Below is a description of the core intervention, and the associated study as carried out in the four second-line countries.

### Core interventions

**Fig. 1:**
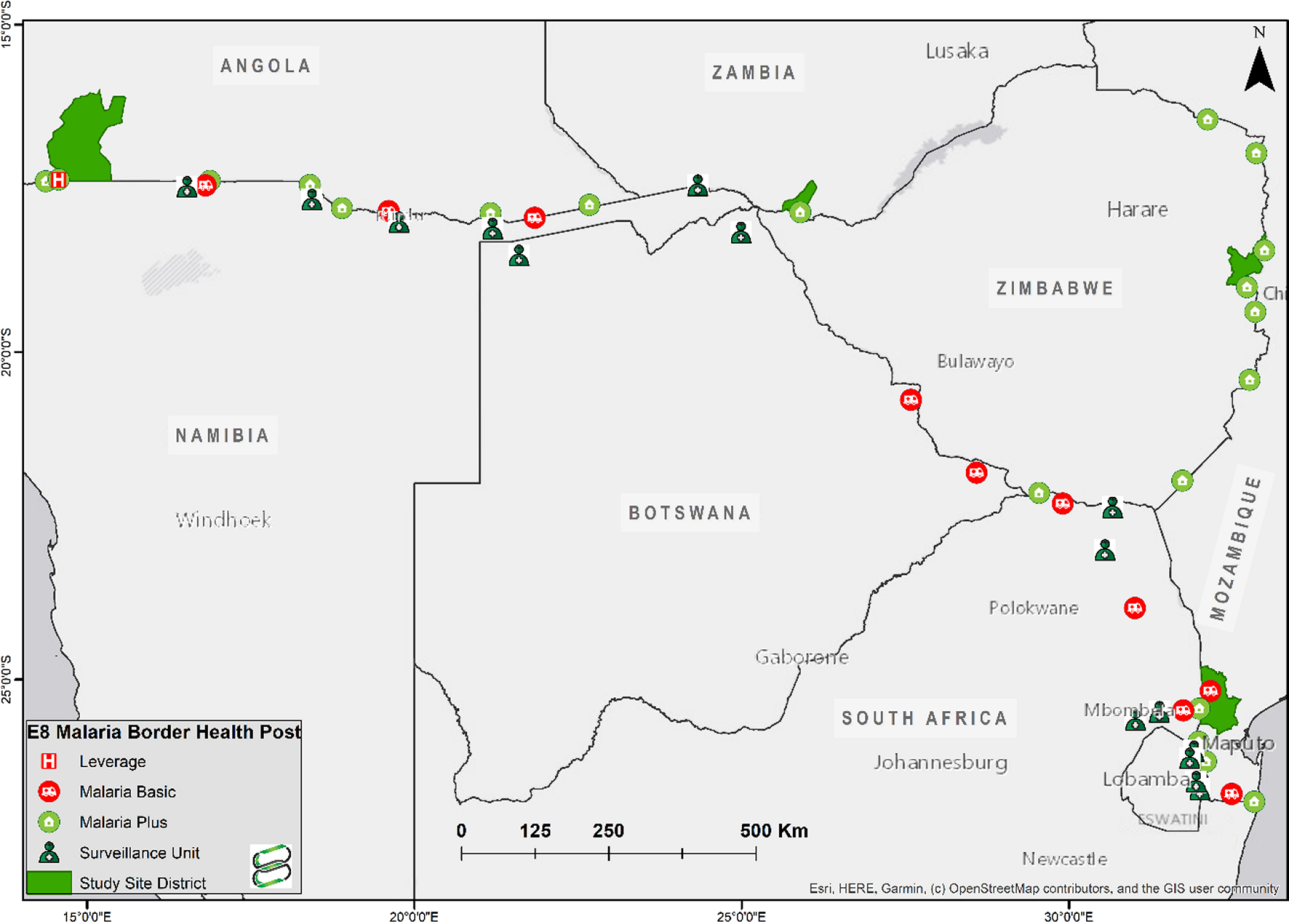
E8 Malaria border health posts, and study sites in second line countries (Source: E8 Secretariat)

In 2017, the E8 with the help of partner organisations established malaria health posts along international frontiers within the region, supported by The Global Fund to Fight AIDS, Tuberculosis and Malaria (see Fig. 1). The locations chosen for border posts followed a desk review and a consultation with national malaria control programs in each country. Candidate locations were border areas, which were underserved in terms of health facility provision, and where there was evidence of a gradient in transmission from one side of the border to the other. The rationale was that borders with different levels of transmission on opposite sides, as evidenced by district health data for malaria, would be prone to significant parasite importation from the high to the low endemicity sides of the border in the absence of adequate malaria services along the border.

The posts offered free testing and treatment to residents of border districts and travellers, and provided outreach services to surrounding communities, as summarized in Table 2.

**Table 2:** Primary models of service delivery for E8 border health posts (Total 46)

Type of post (N)	Malaria plus (21)	Malaria basic (12)	Leverage (1)	Surveillance units (12)
Services offered	Malaria diagnosis and treatment Primary health care package	Malaria diagnosis and treatment	Malaria diagnosis and treatment	Conduct active surveillance along border districts
Structure	Refurbished storage container, with bed for patient	Small tent	Existing health facilities	Vehicle
Target population	Residents and MMPs	MMPs	Residents and MMPs	Residents and MMPs
Mobility level	Static	Mobile	Static	Mobile
Staffing	Nurse, CHW, general hand	Nurse, CHW	Existing health facility staff	Nurse, surveillance/Environmental Health officer

During the 3 years after their formation (2017–2019), a total of 1,207,653 persons were tested for malaria. Of these 71,395 (5.9%) tested positive and were treated for malaria according to country guidelines for first line treatment, in most cases Artemether–Lumefantrine

### Study outcomes

The following outcomes were measured in the study described in this paper: (1) Proportions of respondents receiving timely diagnosis and treatment for febrile illness; (2) Proportions of respondents able to access health facilities for malaria diagnosis and treatment; (3) Proportions of respondents with access to malaria prevention; (4) Respondents’ knowledge of symptoms and malaria prevention, and awareness of border health posts; and (5) Frequency of domestic and international travel.

### Study design and implementation

The study in second line countries, was conducted in one site located in the vicinity of a malaria border post in each of the four countries, selected in consultation with national malaria control programmes, at border crossings with high proportions of mobile populations (Fig. 1). The study population included residents of all ages within a 30 km radius of a selected malaria post.

Using census data, each study area was segmented into geographic clusters of roughly 100–125 households each. Three of these segmented clusters were randomly selected, and 25 households were randomly sampled in each segment to achieve a sample of 75 households per site. An additional 20% contingency was included to allow for non-response.

Written informed consent was sought from the head of each selected household, or from a responsible household member acting as their representative. A structured questionnaire was used to obtain information on knowledge of malaria, attitudes toward and access to malaria posts and existing health facilities, the proportion of residents receiving diagnosis and treatment for febrile illness, practices for malaria prevention, and risk factors for malaria associated with local and international travel. Sections of the questionnaire were adapted from the standard Roll Back Malaria Indicator Survey [11]. Questions were answered by the head of household or their representative on behalf of individuals within a selected household; parents or guardians responded on behalf of children under 18 years of age.

It was assumed that 75 households would need to be selected in each site to achieve a sample size of 300 individuals per site, or 1200 individuals for the four countries combined. In sites where average household size differed from 4 individuals per household, the total number of households was adjusted to achieve the target of approximately 300 individuals. The sample size of 300 per site was pragmatically chosen to match the resources that were available to conduct the study. For questions that were only asked of subgroups of participants, for example proportions seeking treatment out of those who had fever within the recall period, the sample sizes were necessarily smaller.

In Mozambique, Centro de Investigação em Saúde de Manhiça (CISM) conducted the study. In Angola, Zambia and Zimbabwe, the study was carried out by their respective NMCPs. The studies were overseen and coordinated by Elimination 8 Secretariat. Fieldwork was conducted within 6 to 18 months of the implementation of the border posts in each country (Table 3).

**Table 3:** Date of implementation of border health posts and timelines by country

Country	Date when border health post became operational	Timelines of fieldwork
Angola	September 2017	January–February 2018
Mozambique	March 2017	August–September 2018
Zambia	September 2017	June 2018
Zimbabwe	September 2017	June 2018

Ethics approval was obtained from the University of California San Francisco Committee on Human Research (IRB# 17-23221), and the ethics committees of implementing partner organisations in each country. The E8 Research Subcommittee acted as a study steering committee.

The study was funded by The Bill and Melinda Gates Foundation through a grant to the University of California San Francisco. Additional funding for the study was obtained from The Global Fund to Fight AIDS, tuberculosis and Malaria. The funders had no role in the publication of the study.

## Results

Study results are tabulated under the following themes: access to diagnosis and treatment for febrile illness; access to malaria prevention; international and domestic travel; and knowledge of malaria.

### Access to diagnosis and treatment for febrile illness

Fewer than 70% of respondents reported seeking care for febrile symptoms within 48 h of onset of fever amongst adults and children in Angola, adults in Mozambique and children in Zambia (Table 4). A high proportion, but not all of those who presented with fever received a blood test. The highest testing rate was reported in Zimbabwe for both adults and children. Overall, due to neither treatment-seeking nor testing of those who do seek treatment being universal, only about half of those who reported fever had a diagnostic blood test, Zimbabwe being the exception with nearly all those who had fever receiving a test. Nearly all those with a positive test result reported receiving medication.

**Table 4:** Treatment-seeking behaviour and access to diagnosis and treatment: resident respondents seeking treatment for febrile illness (recall < 4 weeks) and respondents who received a blood test for malaria, by country

Indicator	Adults				Children < 18 years			
	Angola	Mozambique	Zambia	Zimbabwe	Angola	Mozambique	Zambia	Zimbabwe
Proportion of respondents who had fever in the last 4 weeks, % (N)	16 (277)	19 (345)	15 (229)	14 (331)	13 (366)	18 (430)	19 (393)	10 (72)
Proportion of respondents with fever within the previous 4 weeks who reported seeking treatment, % (N)	73 (44)	70 (64)	89 (35)	98 (45)	59 (49)	100 (76)	62 (73)	100 (7)
Among those seeking treatment, average number of days from fever onset to seeking treatment, days (N)	1.91 (32)	1 (45)	1.16 (31)	1.18 (44)	2 (29)	0.86 (76)	1.67 (45)	1.71 (7)
Proportion of respondents to seek treatment within 48 h of fever of all those who had fever in the last 4 weeks, % (N)	36 (44)	64 (64)	80 (35)	93 (45)	35 (49)	73 (76)	47 (73)	86 (7)
Proportion of respondents to seek treatment within 48 h of fever of all those who sought treatment, % (N)	50 (32)	91 (45)	90 (31)	95 (44)	59 (29)	73 (76)	76 (45)	86 (7)
Proportion of respondents who were tested with a blood test of all who had fever within 4 weeks, % (N)	52 (44)	50 (64)	54 (35)	93 (45)	51 (49)	66 (76)	47 (73)	100 (7)
Proportion of respondents who were tested with a blood test of all who sought treatment, % (N)	72 (32)	71 (45)	61 (31)	95 (44)	86 (29)	66 (76)	76 (45)	100 (7)
Proportion of respondents who tested positive for malaria of those who were tested, % (N)	39 (23)	16 (32)	0 (19)	83 (42)	52 (25)	12 (50)	6 (34)	57 (7)
Proportion of respondents who received any medication or treatment for malaria of those who tested positive for malaria, % (N)	89 (9)	100 (5)	0 (0)	97 (35)	92 (13)	100 (6)	100 (2)	100 (4)

The numbers in brackets in the first row of the table are the number enrolled at each site

The overwhelming majority of respondents used either a government hospital or other government health facilities as their first choice for seeking treatment for febrile illness (Table 5). Few used private sector facilities including pharmacies, and none reported traditional healers as their first choice for diagnosis of fever. Community health workers were the first choice for seeking diagnosis and treatment for the majority of respondents in Zimbabwe.

**Table 5:** Access to treatment: among those with fever who sought treatment, first choice for seeking treatment, reasons for first choice for seeking treatment, time taken, distance travelled and treatment given, by country (recall < 4 weeks)

Indicator	Adults				Children < 18 years			
	Angola	Mozambique	Zambia	Zimbabwe	Angola	Mozambique	Zambia	Zimbabwe
First choice for diagnosis and treatment for malaria, N	32	45	31	44	29	76	45	7
Government hospital or other government health facility, %	91	91	100	25	76	97	100	100
Private hospital, clinic, doctor, or pharmacy%	6	7	0	0	7	2	0	0
Traditional health practitioner, %	0	0	0	0	0	1	0	0
Community health worker, %	0	0	0	66	7	0	0	0
Other/don’t know, %	0	2	0	9	10	0	0	0
Reasons why first sought treatment at chosen place, N	68	45	31	44	NA	NA	NA	NA
Closest and most convenient, %	69	69	100	91				
Cheapest, %	9	0	0	0				
Has most resources, best reputation, provides best care %	14	18	0	10				
Other/don’t know, %	4	11	0	16				
Time taken to travel to place of diagnosis and treatment, N	32	45^a^	31	44	29	76^b^	45	7
Less than 15 min, %	31	31	74	48	38	25	80	86
15–45 min, %	25	40	19	32	24	46	20	14
More than 45 min, %	44	20	7	20	38	18	0	0
Distance travelled to place of diagnosis and treatment, N	32^c^	45	31	44	29	76^d^	45	7
Less than 2 km, %	41	36	100	80	52	7	91	100
3 to 8 km, %	31	22	0	18	16	34	4.4	0
> 8 km, %	25	42	0	2	32	7	0.4	0

Some percentages do not sum to 100 due to rounding

^a^9% missing data/other

^b^11% don’t know

^c^ 3% don’t know

^d^59% don’t know/other

Convenience and nearness were the predominant reason given for seeking treatment at a given facility, with quality and cost also mentioned in Angola and Mozambique. Most respondents lived within 45 min travel time of the facility where they sought diagnosis and treatment and, probably due to the design of the study, many lived within 15 min of their chosen facility. At the site in Angola, however, many respondents (44% of adults and 36% of children) lived more than 45 min from a place where they would seek health care (Table 5).

**Table 6:** Insecticide treated net ownership and usage, by country

Indicator	Adults			
	Angola	Mozambique	Zambia	Zimbabwe^a^
Proportion of households that own at least one mosquito net, % (N)	65 (132)	87 (333)	96 (202)	49 (198)
Average number of nets owned per households, nets (N)	3.29 (86)	2.5 (289)	2.10 (194)	1.82 (98)
Average number of nets per household occupant, nets (N)^b^	0.44 (643)	0.95 (775)	0.65 (662)	0.44 (403)
Average time since households got their nets, months (N)	2.78 (86)	46 (422)^c^	8.80 (194)	47.74 (98)
Proportion who slept under a net out of all those own nets (household head only), % (N)	91 (86)	71 (289)	89 (194)	70 (98)
Proportion who slept under a bet net out of all respondents, % (N)	59 (132)	62 (333)	86 (202)	35 (198)
Proportion who think nets are effective, % (N)	98 (132)	96 (333)	98 (202)	100 (98)
General bed net condition, N	86	289	194	98
Good (no holes), %	94	72	52	15
Fair (no holes that fit torch battery), %	6	21	26	7
Poor to unsafe (1 or more holes that fit a torch battery), %	0	6	22	78

Questions on nets were only asked of household heads, except in Mozambique where all adult household members were asked

^a^The study area in Zimbabwe is targeted for IRS only, not LLINs

^b^Proportion of households with at least 1 net per 2 persons (universal access) could not be calculated due to limitations of the data

^c^In Mozambique, data were obtained for all nets

### Access to malaria prevention

Net ownership was high in Mozambique (87%) and Zambia (96%), and moderately high in Angola (65%). The lower ownership in Zimbabwe needs to be seen in the context of this site being in an IRS area, which was not targeted for LLIN ownership. In the three sites where LLINs were the primary means of vector control (Angola, Mozambique, Zambia), the number of nets owned per household (of those who owned nets) was above two. Although this figure was highest in Angola, this needs to be seen in the context that a larger proportion did not own any nets in this site. Unfortunately, data on total number of residents per household were not collected and therefore the proportion of households meeting the universal access provision of at least one net per two individuals could not be calculated. Based on an assumption of 4 individuals per household on average, the overall (average) provision of nets was near to or above the universal access target of at least one net per two individuals. In the three sites where LLINs were the main vector control policy, net use was high amongst those who owned nets (71–91%), and moderately high at an overall population level (59–86%). Nearly all respondents thought that nets were an effective means of preventing malaria (> 96%). In the three LLIN sites, the condition of most nets was good or fair.

Reasons for not using a net were a perceived absence of mosquitoes, nets being in poor condition, insufficient numbers of nets or because it was too hot at night (data not tabulated) (Table 6).

Nearly all household heads in Zimbabwe reported previous IRS (96%), more than half did so in Zambia (57%) and 42% in Mozambique. No previous IRS was reported by respondents in Angola (Table 7). In all three of the sites that reported previous IRS this was conducted within the current malaria season. High proportions of respondents believed that IRS was effective for preventing malaria in locations that also reported high coverage (Zambia and Zimbabwe) but only around 50% thought that IRS was effective in the other two sites.

**Table 7:** Reporting of indoor residual spraying (IRS) by country

Indicator	Angola	Mozambique	Zambia	Zimbabwe
Proportion of households ever sprayed with insecticide, % (N)	0 (132)	42 (324)	57 (202)	96 (198)
Average time since last spraying, months n, (N)	0 (0)	2.4 (136)	2.59 (116)	2.95 (192)
Average number of times the household was sprayed in past 12 months, n (N)	0 (0)	1.2 (136)	1.20 (116)	1.02 (192)
Proportion of respondents that slept in a sprayed structure last night, % (N)	0	82 (136)	85 (116)	83 (192)
Proportion of respondents who think IRS prevents malaria, % (N)	50 (277)	47 (175)	80 (229)	99 (198)

### International and domestic travel

In the sites in Angola and Mozambique > 40% of respondents said they travelled during the previous 3 months. Travel was both domestic and across international borders. The reasons for domestic travel were mainly personal in Mozambique, i.e., visiting relatives, whereas the main reason for international travel was of an economic nature (for trade or shopping). In Angola 25% of international travel was to seek medical treatment. Sleeping outside whilst travelling was common for adults (ranging from 17 to 43%), particularly for Angolans and Mozambicans. Small numbers reported using any protective measures except in Zimbabwe where 39% reported using protective measures. Bed nets were the most common protective measure used, followed by the use of repellents. Chemoprophylaxis was hardly ever used (Table 8).

**Table 8:** Local and international travel by country (recall < 3 months)

Indicator	Adults				Children < 18 years			
	Angola	Mozambique	Zambia	Zimbabwe	Angola	Mozambique	Zambia	Zimbabwe
Proportion of respondents who travelled and stayed at least one night outside the district of residence in last 3 months, % (N)	43 (277)	46 (352)	8 (229)	16 (331)	9 (366)	34 (340)	2 (393)	1 (72)
Average number of times respondents travelled within country in last 3 months, n (N)	1.5 (120)	2.3 (161)	1.33 (18)	1.69 (54)	8 (33)	4 (114)	1 (6)	1.0 (1)
Average number of times respondents travelled internationally in last 3 months, n (N)	1.4 (120)	1.8 (161)	0.61 (18)	3. 67 (54)	20 (33)	3 (114)	0 (6)	1.0 (1)
Reason for domestic travel during last trip, N	11	117	11	3	6	108	6	0
For work, %	0	5	18	0	0	0	0	0
For school, %	0	2	0	0	16	56	0	0
To visit family or friends, %	73	53	18	33	16	13	0	0
Marriage, funeral, holiday, %	0	16	27	0	33	18	100	0
Seeking treatment, %	0	2	18	0	33	2	0	0
Trading/selling/shopping, %)	27	8	18	67	0	1.9	0	0
Cattle herding, religious activities, other, don’t know, (%)	0	15	0	0	0	9	0	0
Reason for international travel during last trip, N	109	44	7	51	27	6	0	1
For work, %	4	2	0	12	0		0	0
For school, %	1	0	0	0	30	50	0	0
To visit family or friends, %	19	20	0	33	7	33	0	0
Marriage, funeral, holiday%	10	5	0	10	56	0	0	100
Seeking treatment, %	25	0	0	0	7	0	0	0
Trading/selling/shopping, %)	23	52	100	29	0	0	0	0
Cattle herding, religious activities, other, don’t know, (%)	18	20	0	28	0	17	0	0
Proportion of respondents who slept outside at least 1 day on trip, % (N)	43 (120)	43 (161)	17 (18)	26 (54)	30 (27)	39 (114)	0 (6)	0 (1)
Proportion of respondents who travelled and used protective measures against malaria, % (N)	7 (120)	21 (152)	22 (18)	39 (54)	4 (27)	20 (114)	0	1
Protective measures used by respondents when sleeping outside during their most recent trip, N	8	33	4	21	1	23	0	1
Bed nets, %	75	64	50	71	0	70	0	100
Repellent, %	0	12	50	10	100	13	0	0
Chemoprophylaxis/medicine, %	0	3	0	0	0	0	0	
Covering clothing, %	0	6	0	24	0	0	0	0
Aerosol sprays, %	13	6	0	0	0	0	0	0
Coils, fire, smoke, other/don’t know, %	13	34	0	19	0	17	0	0

#### Knowledge of malaria

Mosquito bites were correctly identified as a potential cause for malaria by around 90% of respondents. Fever was named by a majority as a symptom of malaria. In some sites a larger proportion mentioned other symptoms such as chills and headaches as symptoms of malaria. Death was recognized by most as the worst possible outcome of malaria, although a minority (in Angola) said body weakness was the worst outcome. Very high proportions thought that mosquito nets were an effective means of protection. Considerable proportions of respondents mentioned incorrect prevention methods, such as cutting grass and burning of leaves. Not many respondents identified spraying the house with insecticide as a protective measure even in countries where it is the main means of vector control. This may be because respondents were thinking of measures, they themselves could undertake, rather than protective interventions in general (Table 9).

**Table 9:** Knowledge about malaria and its prevention by country

Indicator	Adults			
	Angola	Mozambique	Zambia	Zimbabwe
Respondents’ knowledge of causes of malaria, N	275	296	229	329
Mosquito bites, %	95	87	93	94
Respondents’ knowledge of symptoms of malaria, N	275	296	229	329
Fever, %	67	65	75	48
Feeling cold/chills/shakes, %	20	53	76	79
Headache, %	4	70	69	73
Nausea and vomiting, %	1	22	60	51
Diarrhoea, %	0	10	7	22
Body ache or joint pain, %	3	40	30	51
Respondents’ knowledge of worst outcome if malaria is left untreated, N	275	296	229	329
Fever, %	1	1	0	1
Body weakness, %	13	1	0	0
Death, %	77	86	95	90
Respondents’ knowledge of how to protect yourself from malaria, N	275	296	229	329
Sleep under a mosquito net, %	75	71	90	74
Sleep under insecticide treated net, %	11	19	6	10
Use mosquito repellent spray, %	0	14	10	11
Avoid mosquito bites, %	1	15	3	3
Take preventive medication, %	4	2	8	1
Spray house with insecticide, %	0	8	10	11
Use mosquito coils, %	0	39	4	13
Cut grass around the house, %	0	17	11	50
Fill in puddles of stagnant water, %	0	18	7	53
Keep house surroundings clean, %	0	47	11	40
Burn leaves, %	1	11	2	19
Other/don’t know, %	5	30	19	27

## Discussion

Cross-border malaria has been shown to be a major obstacle to malaria elimination [5, 9, 12]. To reduce the level of malaria importation from high to low burden countries in Southern Africa, the E8 made a major investment in setting up malaria border health posts along key borders in the region [2]. Recognizing the importance of border malaria in the quest for malaria elimination, a strategy for district level elimination has been adopted in border districts on both sides of borders separating high and low burden countries in the region [3]. To achieve the goals of reduction in cross-border importation and local elimination in border districts, comprehensive surveillance of malaria in border areas is essential. This study has provided a snapshot of access to malaria prevention, diagnosis and treatment and malaria related knowledge and behaviour in four border districts in so called second line (high burden) countries in the region.

Routine testing for malaria of febrile patients is a cornerstone of case management in settings where malaria is endemic. This study found that amongst border residents care-seeking for febrile illness was generally high. In Mozambique and Zimbabwe all children were reported to have sought care for the most recent febrile episode, but care seeking for children was low for children in Angola and Zambia. Not seeking care was mainly due to respondents judging that the fever episode did not warrant seeking care, but care-seeking may also be dependent on how accessible the nearest health care is. For this study survey sites were chosen to be within 30 km of a border post; hence the majority of respondents said that they lived within 45 min of the facility where they sought diagnosis and treatment. For most respondents the first and most convenient choice for seeking treatment was a government hospital or health facility. In the Zimbabwean site community health workers were most frequently mentioned as the preferred choice for seeking diagnosis and treatment for fever. Given that health care was generally accessible but a minority of respondents did not seek care when they or their children experienced fever, there appears to be a need for more effective messaging to convey the risk of serious disease and death if fever episodes are left undiagnosed and hence untreated. At the Angolan site many residents required more than 45 min to reach health care, which may explain the lower level of care-seeking within 48 h of fever at this site. Of concern is the finding that a proportion of respondents said they did not receive a blood test when they presented with fever. There is, therefore, a need for stronger direction to providers to adhere to testing guidelines in areas where malaria infection cannot be ruled out, even when malaria cases are rare. Supply chain management to prevent RDT stock outs may also require improvement to ensure these do not lead to testing not being done. The clear exception in this regard was the site in Zimbabwe where near universal testing of all those presenting with fever was reported. It is noteworthy that care was mostly provided by community health workers in Zimbabwe. This group of health workers provided high levels of adherence to guidelines when presented with patients experiencing fever; they were clearly the first choice for seeking diagnosis and treatment for the majority of respondents in Zimbabwe, and their presence in communities resulted in high proportions of respondents seeking treatment within 48 h of the onset of fever. This model of providing first line health care for malaria is, therefore, an example that other countries should consider adopting.

The study did not include testing respondents for malaria infection, but respondents were asked the result of the blood test at their last clinic attendance, if they were tested. Many reported a positive result (Table 4), indicating a high malaria burden of malaria, and potential for cross-border transmission given the high incidence of reported travel across borders. This underscores the importance malaria border health posts in reducing the burden and transmission of malaria in border areas, and across borders.

A positive finding from this study is that provision of protection against malaria through either mosquito nets or IRS was generally high. High proportions of households owned nets, particularly in Mozambique and Zambia, and those who owned nets, mostly slept under a net the night before the survey. Overall net provision was close to or exceeded the universal coverage criterion of one net per two persons where nets were the primary vector control intervention. A high proportion (> 76%) of nets were in good or fair condition. There was near universal acceptance of the effectiveness of nets to prevent malaria. For a minority, the obvious barrier to net usage was not owning a net.

In all sites adult respondents reported travel over the previous 3 months both within country, and across borders. Travel frequency was particularly high at the Angolan and Mozambican sites. The Angolan site had the highest proportion of cross border travellers, of whom a significant proportion travelled to seek medical treatment. This was mostly into Namibia (data not tabulated) and underscores the importance of malaria border screening to reduce the importation of malaria into low burden countries nearing elimination. Visiting friends or relatives was a common reason for travel, reflecting close ties between communities across borders in the region. A significant proportion travelled for economic reasons such as trading or shopping.

Sleeping outside whilst travelling was commonly reported, with many doing so without any protection against malaria. This represents an obvious gap in malaria protection for a group that are at high risk of malaria infection. Some reported using nets while sleeping outdoors, but in countries where the provision of IRS rather than nets is standard policy, there is a need to make provision for travellers in some other form, be it through chemoprophylaxis or repellents, or making mosquito nets available for the particular purpose of providing protection whilst travelling.

This investigation had important limitations. (1) The descriptive nature of the design had no comparator as it only provided data from communities in the vicinity of a border posts and there were no data on the period preceding the establishment of border posts. Nevertheless, it provides an insight into the provision of malaria services in these areas. (2) MMPs were not surveyed in the study. (3) The survey sample size was relatively small and confined to one locality in each country. Findings should, therefore, be regarded as illustrative, rather than statistically representative of the entire border. (4) The surveys represent snapshots in time, and not all were conducted at the same time in each country. Consequently, some results may have been confounded by the time of fieldwork in the seasonal malaria cycle.

### The overall conclusions were

- In some settings, a proportion of border residents did not receive a blood test when experiencing fever, either because they did not access health care, or because they were not tested when presenting with fever. Whilst most providers carried out blood tests when individuals presented with fever, there were exceptions that are cause for concern and remedial action.
- Community health workers can play a key role in ensuring convenient and timely access to malaria diagnosis and treatment. This is a cost-effective option that should be considered in border districts where access is poor, and malaria infections go undetected and lead to cross-border transmission.
- In general lack of access to health care due to distance or cost or mistrust of the provider was rare.
- There was a high level of correct knowledge of causes, symptoms and prevention of malaria.
- Nearly all those who reported a positive blood test result received medication at the place where they sought care.
- A majority, but not all border residents had access to primary prevention against malaria through either LLINs or IRS. Although overall provision of LLNs was high, a proportion of households did not own any nets, even in sites where this was the main form of vector control.
- Bed nets were overwhelmingly regarded as an effective method of preventing malaria.
- Border residents travelled frequently both within their own country and across borders; some cross-border travel was for the purpose of seeking healthcare.
- Sleeping outside whilst travelling was common and mostly without any protection against malaria; there was a clear gap in the provision of malaria prevention for this group.

Given the high level of travel by border residents and high levels of infection in many border areas, this study demonstrated the importance of border health facilities in providing access to malaria services. Prevention needs to be improved, particularly for people who travel and sleep outdoors. Community health workers can play a key role in providing access to testing and treating individuals with malaria symptoms. Messaging to communities to seek treatment when experiencing fever, and to health care providers to test those presenting with fever, needs to be intensified to ensure that symptomatic infected individuals do not go untreated.

## Data Availability

The datasets generated and analysed during the current study are available from the corresponding author on reasonable request.
